# Iron Overload and Apoptosis of HL-1 Cardiomyocytes: Effects of Calcium Channel Blockade

**DOI:** 10.1371/journal.pone.0112915

**Published:** 2014-11-12

**Authors:** Mei-pian Chen, Z. Ioav Cabantchik, Shing Chan, Godfrey Chi-fung Chan, Yiu-fai Cheung

**Affiliations:** 1 Department of Pediatrics and Adolescent Medicine, The University of Hong Kong, Hong Kong, China; 2 Department of Biological Chemistry, Alexander Silberman Institute of Life Sciences, Hebrew University of Jerusalem, Safra Campus at Givat Ram, Jerusalem, Israel; Indiana University School of Medicine, United States of America

## Abstract

**Background:**

Iron overload cardiomyopathy that prevails in some forms of hemosiderosis is caused by excessive deposition of iron into the heart tissue and ensuing damage caused by a raise in labile cell iron. The underlying mechanisms of iron uptake into cardiomyocytes in iron overload condition are still under investigation. Both L-type calcium channels (LTCC) and T-type calcium channels (TTCC) have been proposed to be the main portals of non-transferrinic iron into heart cells, but controversies remain. Here, we investigated the roles of LTCC and TTCC as mediators of cardiac iron overload and cellular damage by using specific Calcium channel blockers as potential suppressors of labile Fe(II) and Fe(III) ingress in cultured cardiomyocytes and ensuing apoptosis.

**Methods:**

Fe(II) and Fe(III) uptake was assessed by exposing HL-1 cardiomyocytes to iron sources and quantitative real-time fluorescence imaging of cytosolic labile iron with the fluorescent iron sensor calcein while iron-induced apoptosis was quantitatively measured by flow cytometry analysis with Annexin V. The role of calcium channels as routes of iron uptake was assessed by cell pretreatment with specific blockers of LTCC and TTCC.

**Results:**

Iron entered HL-1 cardiomyocytes in a time- and dose-dependent manner and induced cardiac apoptosis via mitochondria-mediated caspase-3 dependent pathways. Blockade of LTCC but not of TTCC demonstrably inhibited the uptake of ferric but not of ferrous iron. However, neither channel blocker conferred cardiomyocytes with protection from iron-induced apoptosis.

**Conclusion:**

Our study implicates LTCC as major mediators of Fe(III) uptake into cardiomyocytes exposed to ferric salts but not necessarily as contributors to ensuing apoptosis. Thus, to the extent that apoptosis can be considered a biological indicator of damage, the etiopathology of cardiosiderotic damage that accompanies some forms of hemosiderosis would seem to be unrelated to LTCC or TTCC, but rather to other routes of iron ingress present in heart cells.

## Introduction

As an essential element for almost all living organisms, iron serves as a critical component in different metabolic processes including oxygen transport and storage, DNA, RNA and protein synthesis, and electron transport [1]. Tight regulation of iron concentrations is required for maintenance of cellular function, while excessive iron leads to generation of oxidative stress by increasing production of reactive oxygen species [2]–[4]. Of the different organs, the heart is particularly vulnerable to iron toxicity [5].

Iron overload cardiomyopathy (IOC) is well documented in patients with β-thalassemia major and is an important cause of morbidity and mortality [6]–[9]. Clinical manifestations include systolic and diastolic ventricular dysfunction, cardiac arrhythmias, and end-stage cardiomyopathy [5], [8], [10], [11]. However, the mechanisms of iron-induced subclinical cardiac dysfunction and end-stage cardiomyopathy remain unclear. Progressive loss of cardiomyocytes, albeit at a low level, through apoptosis is believed to contribute to the remodeling process and ventricular dysfunction in heart failure [12]–[17]. There is, however, a paucity of data on the phenomenon of cardiomyocyte apoptosis and the pathway involved in the setting of iron overload.

Under physiologic condition, iron uptake into cardiomyocytes is mediated through transferrin-transferrin receptor-mediated endocytosis with negative feedback regulatory mechanisms [18]. However, under iron overloading conditions, transferrin becomes saturated and excess plasma iron will present as non-transferrin-bound iron (NTBI), which contributes to the intracellular labile iron pool and the generation of reactive oxygen species [9]. Reported mechanisms of NTBI entry into cardiomyocytes are nonetheless controversial [19]. While some studies have proposed L-type calcium channels (LTCC) to be a major pathway for NTBI entry [20]–[22], others suggest that T-type calcium channel (TTCC) may be the alternative portal of entry [23], [24]. However, direct evidence for possible protective effects of calcium channel blockers against iron-induced cardiomyocyte apoptosis is lacking.

Using HL-1 cardiomyocytes, a spontaneously contracting cardiomyocyte cell line that expresses both LTCC and TTCC molecularly and functionally [25]–[27], together with the real-time technique tracing cellular iron uptake and flow cytometry, we explored (i) the phenomenon of and mechanisms involved in cardiomyocyte apoptosis induced by iron overload, (ii) the effects of LTCC and TTCC blockers on Fe(II) and Fe(III) entry into cardiomyocytes, and (iii) the potential protective effect on iron-induced cardiomyocyte apoptosis by calcium channel blockade.

## Materials and Methods

### Cell culture

HL-1 cardiomyocytes were kindly provided by Prof. W.C. Claycomb (Louisiana State University Health Science Center, New Orleans, LA, USA) who created the cell line [25]. HL-1 cells were established from the AT-1 mouse atrial cardiomyocyte tumor, and can be serially passaged while maintaining contractile phenotype. The cells were grown in culture vessels pre-coated with 0.02% gelatin (Difco, Fisher Scientific, Suwanee, GA, USA) - 5 µg/ml fibronectin (Sigma, St Louis, MO, USA) solution at 37°C in a humidified 5% CO_2_ incubator, maintained in Claycomb Medium (SAFC Biosciences, Sigma) supplemented with 10% fetal bovine serum (Sigma), 0.1 mM norepinephrine (Sigma), 2 mM L-glutamine (Invitrogen, Life Technologies, Grand Island, NY, USA) and penicillin/streptomycin (100 U/ml:100 µg/ml) (Invitrogen). The medium was changed approximately 5 days per week.

### Iron treatment and calcium channel blockade

For calcein green-acetomethoxy (CALG-AM) fluorescent assay, HL-1 cells were seeded at 6×10^4^ cells/well in gelatin-fibronectin coated 96-well black CulturPlate (PerkinElmer, Waltham, Massachusetts, USA). Cells reached around 90% confluence after 24 hr culture. L-type calcium channel blockers including amlodipine (Cipla, India) and verapamil (Abbott, Ludwigshafen, Germany) and TTCC blocker, efonidipine (Sigma), were loaded at 0.1, 1, 10, 100 µM in assay buffer, which consisted of HEPES-buffered saline, pH 7.4 (HBS) supplemented with 0.5 mM probenecid (Sigma), 30 min before iron challenge, and the concentrations were maintained during the assay. FeCl_3_ was loaded at 150, 300, 600 µM with and without 1 mM ascorbic acid in assay buffer, which has been indicated to represent Fe(II) and Fe(III) respectively [24], [28], [29]. Controls (with and without ascorbate) was defined as the conditions without calcium channel blockers and iron.

For flow cytometric assay, HL-1 cells were seeded at a density of 1.5×10^5^ cells/ml in gelatin-fibronectin coated plates. After 24 hr incubation, culture medium was changed into norepinephrine-free medium containing 2% fetal bovine serum, 2 mM L-glutamine and penicillin/streptomycin (100 U/ml:100 µg/ml), and also 150, 300, 600 µM FeCl_3_ with and without 1 mM ascorbic acid for test groups. Calcium channel blockers were pre-loaded at 1 µM 60 min before iron challenge without media change before treatment endpoint. For treatments with iron chelator deferiprone (Apotex, Toronto, Canada), 10 or 100 µM deferiprone was loaded 20 min after iron loading. Blank controls (with and without ascorbate) was defined as the conditions without calcium channel blockers, chelator and iron loading. After 72 hr of incubation, cells in the control group had confluency at around 90%, while cells in iron treatment groups had less. Cells were gently detached by 0.05% Trypsin-EDTA (Invitrogen) for flow cytometric assays.

### CALG-AM fluorescent assay

**Figure 1:**
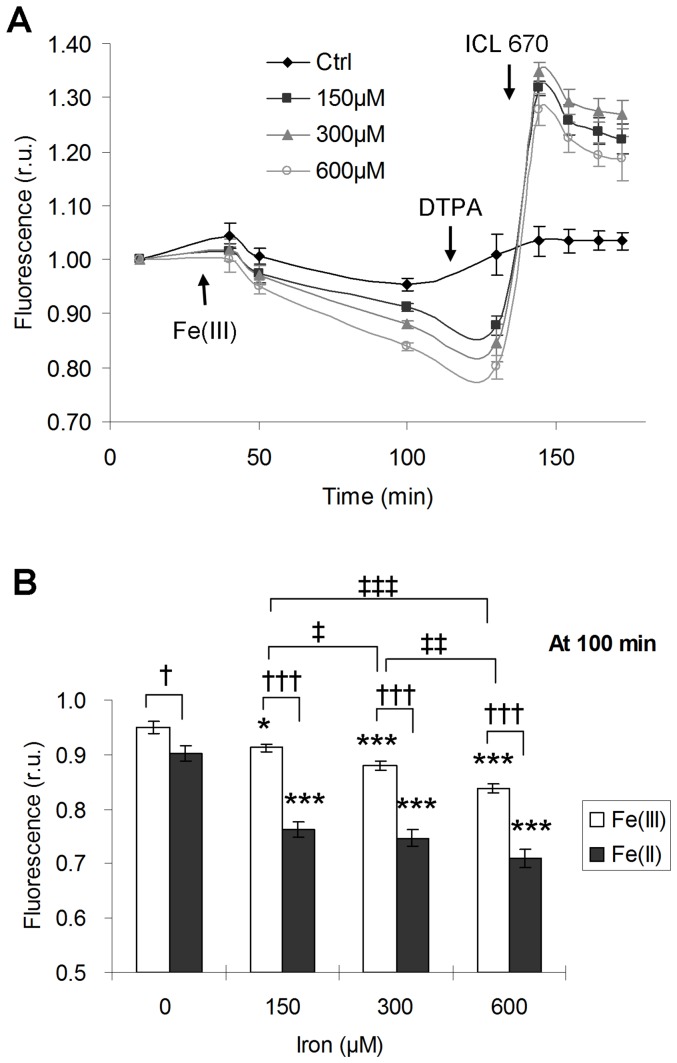
Exogenous iron entered cardiomyocytes in a time- and dose- dependent manner. (**A**) Fe(III) uptake by live HL-1 cells treated at 3 indicated doses, detected by CALG-AM fluorescent assay. Fluorescence intensity was carried out by fluorescent plate reader Fusion. Local average reading at 10 min was set as initial fluorescence level. Each reading at any given time was normalized to the local initial fluorescence level. FeCl_3_ was load at 30 min. Impermeant chelator DTPA was loaded at 115 min; permeant iron chelator ICL 670 was loaded at 136 min. Control was defined as treatment without addition of iron, DTPA and ICL670. (**B**) Fe(III) and Fe(II) uptake at 100 min of the assessment time point indicated in (A), i.e. 70 min after iron loading. FeCl_3_ loaded with ascorbate represented Fe(II) treatment. Both controls with and without ascorbate were shown. *, †, ‡, *p*<0.05; **, ††, ‡‡, *p*<0.01; ***, †††, ‡‡‡, *p*<0.001; * versus respective controls. The results represented as mean ± SEM of five independent triplicate experiments.

To trace iron transport in live HL-1 cells, CALG-AM fluorescent assay was used [30]. Non-fluorescent CALG-AM is converted to green-fluorescent calcein once diffuses into live cells, going through acetoxymethyl ester hydrolysis by intracellular esterases. Cells were exposed to 0.25 µM CALG-AM (Molecular Probes, Life Technologies, Grand Island, NY, USA) at 37°C for 30 min in Claycomb Medium containing 10 mM Na-HEPES (Sigma). Cells were then rinsed with HBS, followed by the perfusion of assay buffer, HBS supplemented with 0.5 mM probenecid, which prevented leakage of anionic fluorescent probes from cells. Calcium channel blockers and ascorbic acid were added simultaneously under the conditions mentioned. Fluorescent intensity was measured using fluorescent plate reader Fusion (Packard, Perkin Elmer Life Sciences, Boston, MA, USA) at excitation/emission wavelength 485 nm/520 nm. Local average reading at 10 min after assay buffer loading was set as initial fluorescence level. FeCl_3_ was loaded at 20 min after the first plate reading (Figure 1A). Calcein was quenched by intracellular labile iron, and hence, the fluorescence intensity was inversely proportional to the level of labile intracellular iron. Iron entry was terminated by adding 100 µM impermeant chelator diethylene-triamine-pentaacetic acid (DTPA) at 115 min after assay buffer loading. Identification of intracellular labile iron was verified by 100 µM permeant iron chelator deferasirox (Exjade, ICL670) at 136 min after assay buffer loading to reverse the calcein-Fe quenching. Control was defined as treatments without addition of calcium channel blockers, iron, DTPA and ICL670. Experiments were performed in triplicate. Each reading at any given time was normalized to the local initial fluorescence level.

### Annexin V/PI assay

Fluorescein isothiocyanate (FITC) Annexin V Apoptosis Detection Kit (Becton Dickinson, Franklin Lakes, NJ, USA) was used according to manufacturer's instructions. Briefly, cells from cultures were collected and washed with cold PBS and then resuspended in annexin V binding buffer. After staining with annexin V-FITC and PI for 15 min at room temperature in the dark, cells suspended in annexin V binding buffer were tested by LSR II flow cytometer (Becton Dickinson). For each measurement, at least 10,000 cells were counted. Flow data were analyzed by FlowJo 8.8.4 (Tree Star). Only single cell events were gated out for analysis.

### Activated caspase-3 assay

FITC Active Caspase-3 Apoptosis Kit (Becton Dickinson) was used according to manufacturer's instructions. Briefly, cells from culture were collected and washed with cold PBS, then fixed and permeabilized in BD Cytofix/Cytoperm solution for 20 min on ice. After washing with BD Perm/Wash buffer, cells were stained with FITC-conjugated anti- active caspase-3 antibody for 30 min at room temperature. With further wash with Perm/Wash buffer, cells suspended in Perm/Wash buffer were tested by LSR II flow cytometer. Flow cytometry was performed as aforementioned.

### JC-1 assay

The mitochondrial membrane potential (Δψ) of HL-1 cardiomyocytes was evaluated by Flow Cytometry Mitochondrial Membrane Potential Detection Kit (Becton Dickinson). JC-1 (5,5′,6,6′-tetrachloro-1,1′,3,3′-tetraethylbenzimidazolcarbocyanine iodide) is a fluorochrome widely used to evaluate the status of Δψ. Mitochondria with normal Δψ increases JC-1 uptake, which leads to the formation of JC-1 aggregates that emit red fluorescence at 590 nm. In depolarized mitochondria, low concentration of JC-1 inside would stay at monomer form, emitting green fluorescence maximally at 527 nm. The staining protocol followed manufacturer's instructions. Briefly, cells were collected and incubated in JC-1 solution for 15 min at 37°C in CO_2_ incubator. After subsequent washes with Assay Buffer, cells were resuspended in Assay Buffer for flow cytometry by LSR II as aforementioned.

### Statistical analysis

Data are presented as mean ± SEM. Statistical analysis was performed using one-way analysis of variance (ANOVA) with post test for multiple comparisons, and unpaired t test for comparisons of two groups by GraphPad Instat 3 (GraphPad Software, Inc., San Diego, CA, USA). A *p*<0.05 was regarded as statistically significant.

## Results

### Exogenous iron entered cardiomyocytes in a time- and dose- dependent manner

To detect intracellular labile iron, iron influx was visualized in real time by tracking the gradual decrease of fluorescence signals in the live HL-1 cardiomyocytes. Within the detection period from 10 to 70 min after iron loading (Figure 1A), we observed iron entering HL-1 cells in a time-dependent manner. With elimination of extracellular iron by addition of the impermeable chelator DTPA, the subsequent addition of permeable chelator ICL670 restored the calcein fluorescence quenched by labile iron significantly, confirming that CALG-AM assay could assess intracellular iron in HL-1 cardiomyocytes effectively.

Based on the difference of uptake rate at 70 min after iron challenge with or without ascorbate, Fe(II) was found to be significantly more permeable than Fe(III) (*p*<0.001) (Figure 1B). Fe(III) showed a dose-dependent acquisition at 150, 300, 600 µM loading. In contrast, Fe(II) achieved a near plateau loading at 150 µM (Figure 1B).

### Iron loading induced cardiomyocyte apoptosis

**Figure 2:**
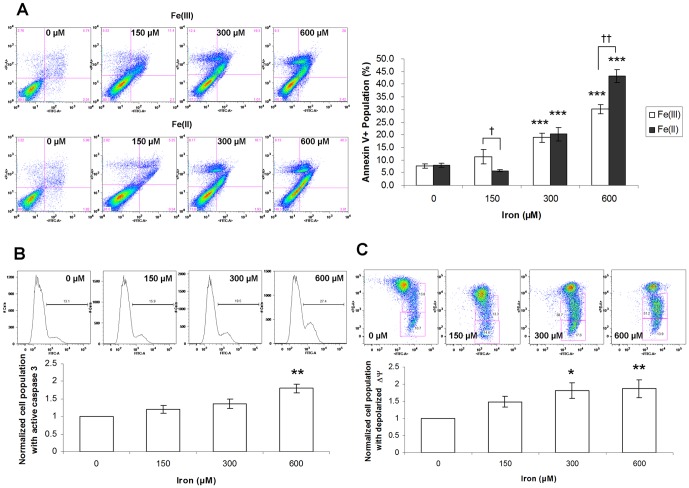
Iron overload induced cardiomyocyte apoptosis. HL-1 cells were treated with Fe(III) and Fe(II) for 72 hr, followed by (**A**) annexin V/PI flow cytometry assay, (**B**) active caspse-3 flow cytometry assay, and (**C**) JC-1 flow cytometry assay. *, †, *p*<0.05; **, ††, *p*<0.01; ***p<0.001; * versus respective controls. The results represented as mean ± SEM of five to six independent experiments.

Annexin V/Propidium Iodide (PI) flow cytometric assay was used to quantify the amount of apoptosis. Cells positive for annexin V but negative for PI represented those undergoing early apoptosis, while cells stained positive for both annexin V and PI represented the population undergoing late apoptosis or necrosis [31], [32]. By quantifying the percentage of total annexin V positive cells (lower and upper right quadrant in the representative flow cytometry charts as shown in Figure 2A), we found a dose-dependent increase in apoptotic cell population when HL-1 cells were treated with FeCl_3_ with or without ascorbic acid for 72 hr (Figure 2A) (pH of each condition changed within 7.4–7.8). Such increase in apoptosis was noted in cells treated with concentrations of FeCl_3_ at ≥300 µM (*p*<0.001). At the concentration of 600 µM, Fe(II) induced significantly more apoptosis than Fe(III) (*p*<0.01).

To further define the underlying apoptotic mechanism of iron overload on HL-1 cardiomyocytes, caspase-3 activity and mitochondrial membrane potential change were also assessed. In line with the findings of annexin V/PI assay, iron overload induced a dose-dependent activation of caspase-3 (Figure 2B) and alteration of mitochondrial membrane potential (Figure 2C), which suggested an involvement of the intrinsic apoptotic pathway.

### High-dose LTCC but not TTCC ameliorated Fe(III) entry under condition of iron load

**Figure 3:**
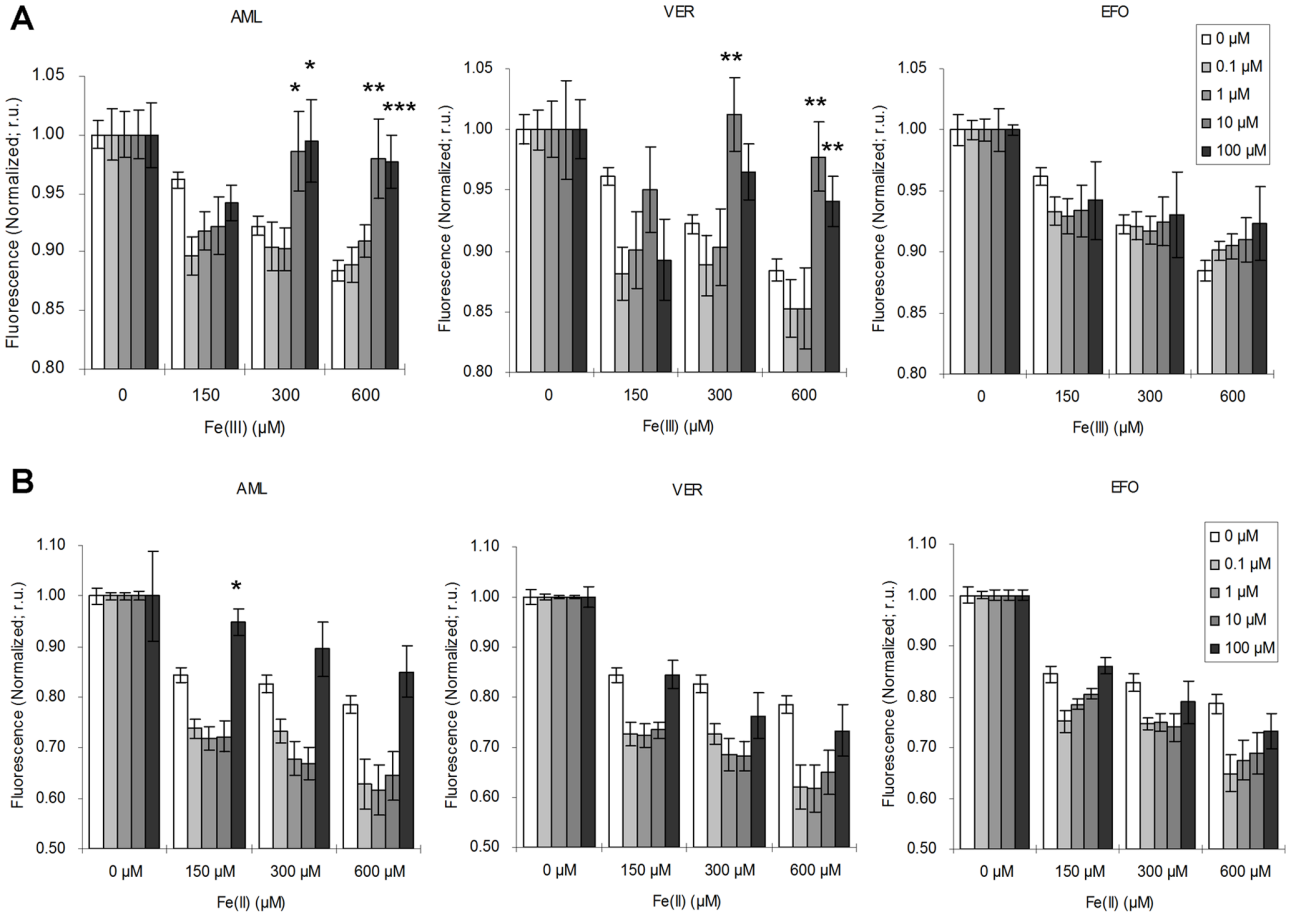
Iron blockade effects of LTCC and TTCC blockers on iron-overloaded cardiomyocytes. In this CALG-AM fluorescent assay, HL-1 cells were pretreated with LTCC blockers, amlodipine (AML) and verapamil (VER), and TTCC blocker, efonidipine (EFO), at logarithmic scale from 0.1 to 100 µM. 3 indicated doses of Fe(III) (**A**) and Fe(II) (**B**) were loaded 30 min after blocker treatment. Fluorescence readings were at 70 min after iron loading. Fluorescence signal changes were normalized to respective negative controls of each treatment arm. *p<0.05; **p<0.01; ***p<0.001. The results represented as mean ± SEM of four independent triplicate experiments.

The potential roles of LTCC and TTCC for iron entry into HL-1 cardiomyocytes were evaluated using CALG-AM fluorescent assay, with treatments with LTCC blockers, amlodipine and verapamil, and TTCC blocker, efonidipine, at 30 min prior to iron loading. The blockade effects for Fe(III) (Figure 3A) and Fe(II) (Figure 3B) treated at 150, 300, 600 µM were assessed at logarithmic increments of calcium channel blocker concentrations from 0.1 to 100 µM. The time point of assay was at 70 min after iron loading, which was approximately 100 min after administration of different calcium channel blockers. Fluorescent signal changes were normalized to respective negative controls of each treatment arm.

Compared with the increase in iron entry into cells under Fe(III) treatment alone with decreased fluorescent signals, pretreatment with 10 to 100 µM of amlodipine and verapamil significantly increased normalized fluorescent signals (Figure 3A). The effect was more pronounced with 300 µM and 600 µM than 150 µM of Fe(III) load. These findings suggested blockade of Fe(III) entry by both LTCC blockers. However, efonidipine did not exert significant blocking effect on iron entry in Fe(III) overload.

### Trend of LTCC and TTCC blockade of Fe(II) entry

With regard to Fe(II) loading condition, increased trends of fluorescent signals were observed with increased LTCC and TTCC blockade (Figure 3B). However, statistical significance was only found with pretreatment using 100 µM amlodipine.

### Calcium channel blockers could not salvage cardiomyocytes from iron-induced apoptosis

**Figure 4:**
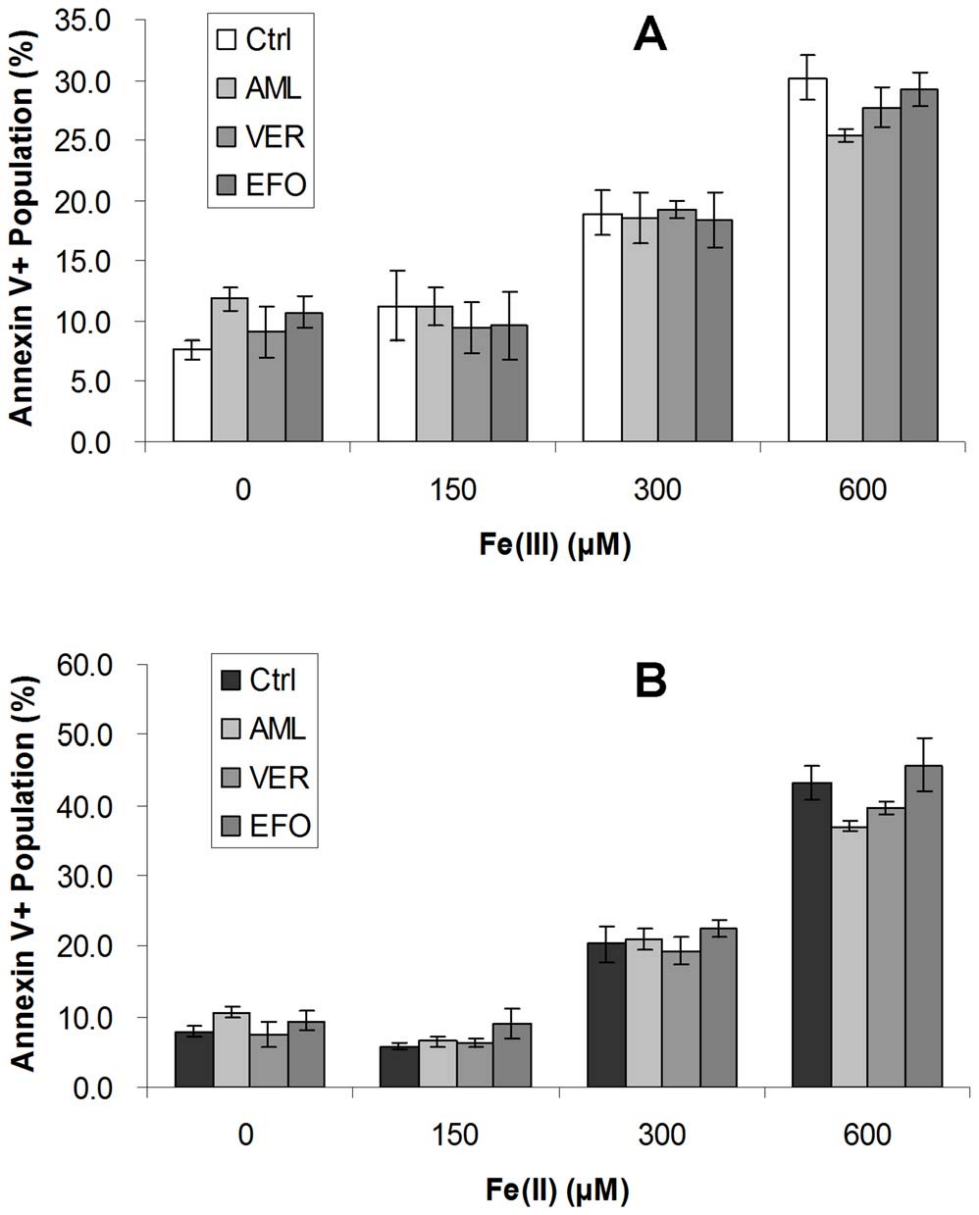
Calcium channel blockers could not salvage HL-1 cells from iron overload induced apoptosis. HL-1 cells were pretreated with LTCC blockers AML or VER, and TTCC blocker EFO for 1 hr, followed by Fe(III) (**A**) and Fe(II) (**B**) loading for 72 hr. Controls were defined as treatments without blockers. Apoptosis was determined by annexin V/PI flow cytometry assay. Total annexin V positive cell portion was counted. The results represented as mean ± SEM of three independent experiments.

To further explore whether calcium channel blockade could reduce HL-1 cardiomyocyte apoptosis induced by iron overload, annexin V/PI assay was performed on HL-1 cells loaded with Fe(III) and Fe(II) at different concentrations, with pretreatment of LTCC and TTCC blockers at a concentration of 1 µM. There was no significant decrease in apoptotic cell population, whether loaded with Fe(III) (Figure 4A) or Fe(II) (Figure 4B).

**Figure 5:**
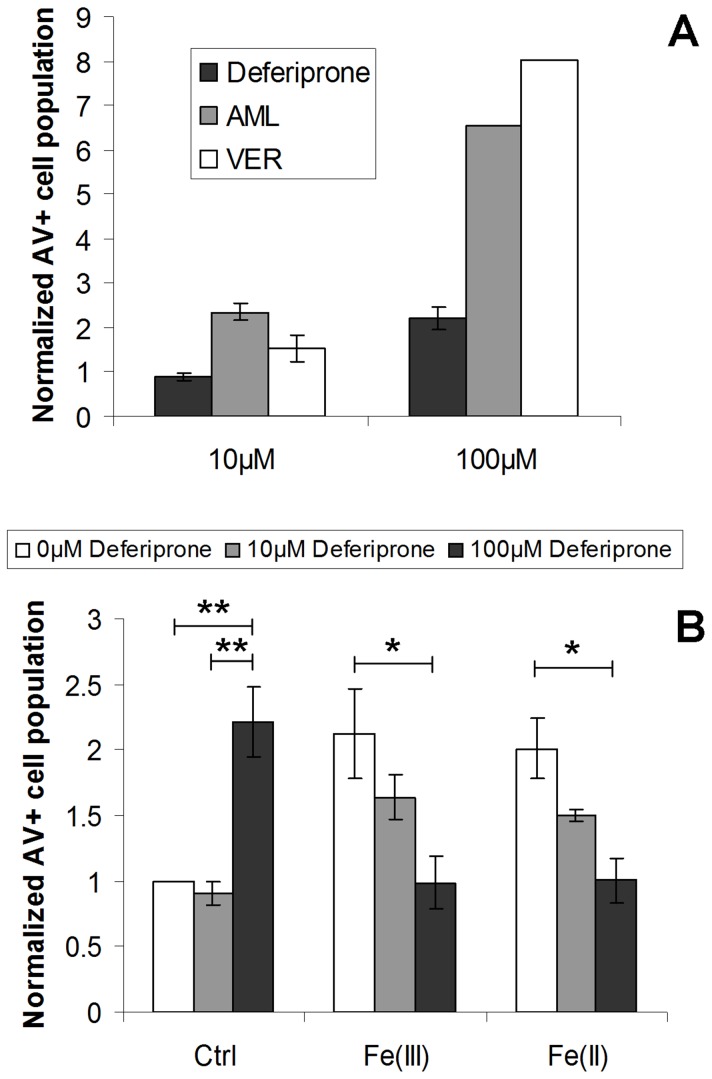
Cellular toxicity of LTCC blockers and the comparison with deferiprone. (A) Apoptotic effects of 10 or 100 µM AML, VER and deferiprone on HL-1 cells for 72 hr were assessed by annexin V/PI flow cytometry assay. (B) HL-1 cells were challenged with 300 µM Fe(III) or Fe(II), followed by treatments of 10 or 100 µM deferiprone 20 min after iron loading. Apoptosis was determined after 72 hr incubation by annexin V/PI assay. Data were shown as total annexin V positive cell portion with normalization to respective negative controls. * *p*<0.05; ** *p*<0.01. The results represented as mean ± SEM of three independent experiments, except 100 µM AML and VER (n = 1).

The findings suggested that calcium channel blockers at this concentration had no protective effects on HL-1 cells against iron-induced apoptosis. However, at the doses of 10 µM or 100 µM, amlodipine or verapamil, which showed significant iron blockade effect on HL-1 cells (Figure 3), appeared to have high cellular toxicity (Figure 5A). Pretreatment of TTCC blockers in iron treated HL-1 cells led to similar or even worse effects.

By contrast, the commonly-used iron chelator deferiprone induced less toxic effect under non-iron overloaded condition (Figure 5A) and further showed protective effect on iron-induced apoptosis of cardiomyocytes (Figure 5B).

## Discussion

The present study shows that i) iron induces apoptosis of HL-1 cardiomyocytes via the mitochondria-mediated caspase-3 dependent pathway, ii) blockade of LTCC but not TTCC prevented Fe(III) but not Fe(II) entry under iron overload condition and (iii) blockade of neither LTCC nor TTCC could salvage the cultured cardiomyocytes from iron overload induced apoptosis.

### Iron-induced cardiomyocyte apoptosis

The levels of plasma NTBI in thalassemia patients under iron overload are variable, with an estimation suggested to be 0-25 µM [33]. For the proof of principle, comparable iron concentrations as previously reported were used in the current *in vitro* study [24], [34]. The apoptotic effect of iron overload on HL-1 cells and its involvement of mitochondria-dependent pathway were suggested by the findings of increase in phosphatidylserine exposure, increased caspase-3 activity, and a dose-dependent drop on mitochondrial membrane potential in iron-overloaded HL-1 cells. Our results are in agreement with the *in vivo* studies suggesting the cardiac apoptotic effect of iron overload on mice [20] and gerbils [35] as revealed by increased nucleic DNA fragmentation and caspase activity. Although other study suggesting the necrotic effect of iron overload on cardiomyocytes [36], more evidences will be of interest to the further mechanism behind, including the postulated cross link between apoptosis and necrosis in series or parallel [37], as well as the differences among experimental models.

### Fe(II) and Fe(III) entry into cardiomyocytes

As both redox states of iron have been shown to form cardiac iron deposit [28], our study explored both ferric and ferrous irons. The results agree with those reported previously regarding the more permeative nature of ferrous iron, which is maintained with ascorbate as a reducing agent [24], as evaluated by kinetic parameters [28], [38]. Previous studies have implicated either the LTCC or TTCC as the main candidate for NTBI entry into cardiomyocytes. The controversies have in part been related to different models and methods used.

### The effect of LTCC blockade on iron entry

Calcium channels play an important role in myocardial contractility and remain open for long duration (>400 ms) in each contraction cycle [39]. Except for the primary transport of Ca^2+^, LTCC also facilitate transport for many other divalent cations including Fe^2+^, Co^2+^ and Zn^2+^ [22], [40], [41]. Previous studies suggest that LTCC is the major portal for iron uptake into cardiomyocytes in IOC [20]–[22]. For a further mechanism, we assessed the role of LTCC in iron-overloaded cardiomyocytes by the real-time approach.

Our results showed significant reduction of ferric iron ingress by both LTCC blockers at higher doses of iron treatment, 300 µM and 600 µM, but not at lower dose of iron at 150 µM. This phenomenon implicated the classic concept of iron delivery through transferrin at lower dose of iron treatment [9], while confirming the blockade effect from LTCC blockers toward excessive iron, Fe(III) from this result, uptake into cardiomyocytes [20]–[22]. It is worth noting, however, that LTCC blockers displayed their iron blockade effect only at concentrations of 10 and 100 µM, higher than the therapeutic serum levels of 0.1 to 1 µM [42], [43]. Hence, the clinical translation of the use of LTCC blockers to prevent iron-induced cardiotoxicity remains uncertain.

It is widely recognized that the promiscuous property of LTCC for the transport of other metals is limited to divalent, but not trivalent cations [22], [40], [41], [44]. Interestingly, our data indicated a significant reduction of Fe(III) uptake, but only a trend to reduce Fe(II) uptake, at the presence of LTCC blockers. Together with evidence that a reduction of Fe(III) is required for NTBI uptake into cardiomyocytes [22], [28], it raised the possibility that LTCC blockers achieve the effect on NTBI blockade not by stopping Fe(II) entry directly but through alternative mechanism. Recent studies provide an alternative explanation on the role of LTCC in NTBI entry. LTCC has been shown to contribute to the activation of endocytotic machinery in neuronal cells [45]. Interestingly, endocytosis has also been demonstrated to be a possible pathway for macromolecule-associated NTBI uptake into various cell types including cardiomyocytes [38], [46]. As LTCC blockade interferes calcium-induced endocytosis, a subsequent interruption of Fe(III) uptake via such pathway can be a possible speculation.

### The effect of TTCC blockade on iron entry

With abundant expression in embryonic cardiomyocytes, and subsequent suppression shortly after birth [47], TTCC has been shown to reappear in murine hearts with pathological abnormalities including hypertrophy [48], myocardial infarction [49] and also thalassemia [23], [24]. Using efonidipine, the TTCC blocker, Kumfu et al. shows effective blockade of iron uptake both *in vitro* and *in vivo* using the thalassemic mice model, together with the protection effects as assessed *in vivo*, while LTCC blockers appeared inferior [23], [24]. However, in our present experimental model, with pretreatment of efonidipine, uptake of neither Fe(II) nor Fe(III) was significantly decreased in iron-overloaded HL-1 cardiomyocytes, implicating an insignificant role of TTCC in HL-1 cells for excessive iron uptake.

### Differences in study models

The mechanisms and portal of iron entry into cardiomyocytes under iron overload condition have been controversial, in part being related to differences in experiment approaches, types of iron load models, and the nature of cardiomyocytes explored. In the present study, immortalized HL-1 atrial myocytes were used, which have the advantages of being the only cardiomyocyte cell line currently available that continuously divides and spontaneously contracts while retaining a differentiated adult cardiac phenotype [25], [26]. Apart from the superior cardiac properties and cell purity compared with isolated primary cardiomyocytes, HL-1 cells express, from molecular and functional regards, both LTCC and TTCC *in vitro* [27]. In addition, atrial myocytes may provide a model for the study of cardiac iron toxicity, given that atrial dilation and dysfunction have been reported to be earlier markers than depressed ventricular function of cardiac iron toxicity in patients with thalassemia major [50].

### LTCC blockade and cardiomyocyte apoptosis

For the therapy of IOC, protection of iron overload induced cardiac apoptosis is apparently crucial beyond the maintenance of regular iron metabolism. Such protection effect was presented in our *in vitro* study by deferiprone, the effective iron chelator commonly used in current clinical practice [51]. However, in our assessment, none of the calcium channel blockers showed significant protection effect on iron overload induced apoptosis, though LTCC blockers, in particular amlodipine, presented slight protection at 600 µM of ferric or ferrous iron challenge. This result is to a certain extent contrary to the previous finding that amlodipine and verapamil attenuate cardiac apoptosis in iron-overloaded mice evaluated by TUNEL assay [20]. One possible explanation is that NTBI initiates apoptosis of cardiomyocyte prior to its entry through cell membrane; and for the *in vivo* model, apart from the effect on NTBI blockade, it cannot rule out the possible contribution from the impacts of LTCC blockers on other physiological conditions which subsequently reduce such iron induced apoptosis. Furthermore, the different susceptibility to iron overload between atrial and ventricular cardiomyocytes should also be taken into consideration [52]. Despite the demonstrable ability of LTCC blockers to inhibit iron ingress into the cytosol of cardiomycytes, their apparent failure to protect them from apoptosis might be due to various properties associated with iron traffic within cells, particularly between cytosol and into mitochondria. As shown earlier [38], [53], [54], a major fraction of exogenously added iron can access mitochondria, by mechanism that seemingly by-pass the labile iron pool, which is sensed by the calcein probe, and it can even be refractory to some intracellular chelators [38]. While those features would imply that LTCC might provide a path for NTBI entry into cardiomyocytes, they also indicate that such paths might not be relevant for trafficking iron across cytosol to mitochondria, particularly in the pathophysiological context. Consequently, although the prevention of iron ingress into cardiomyocytes was observed in treatment with LTCC blockers at higher doses, due to their toxicity, at least shown *in vitro*, further studies would be of importance for their protective roles in iron-overloaded cardiomyocytes, and also for a better understanding of the etiology of IOC.

### Clinical implications

Apoptosis is rare in normal human heart. In all reported cases, including those in failing hearts, apoptosis levels are substantially lower than 1% as revealed by TUNEL assay [55]. Due to the poor regenerative capacity of cardiomyocytes, a constant, albeit low, level of apoptosis can have serious consequence. Apart from limited studies showing the potential anti-apoptotic effect of deferasirox [35] and taurine [4] in myocardium of iron-overloaded murine model, little is known about the anti-apoptotic approach for iron overload. Further studies on the mechanism of iron induced apoptosis would provide novel targets for advanced therapy against IOC.

### Limitations

Several limitations to this study warrant discussion. Firstly, the findings of the present *in vitro* study may reflect perhaps a relatively acute effect of iron load on cardiomyocytes. Ideally, the experimental protocols should be extended to longer duration with lower iron levels. However, given the technical constraints including the confounding influence of cell proliferation with prolonged culture on fluorescent assay of iron entry and the need for medium change with alteration in iron concentrations, we have elected to adopt the current methodology. With regard to animal studies, previous works have been done on mouse [20], [24] and gerbil [35], which mimic the effect of chronic iron overload better, although results remained controversial. Secondly, we have not assessed the effects of calcium channel blockade on cellular beating in the present study. Calcium channel blockade may reduce beating rate or cause cessation of cardiomyocyte contraction *in vitro* [56], [57]. Nonetheless, LTCC and TTCC have been shown to remain functional in HL-1 cells without apparent contraction [27], [58]. The effect of cardiomyocytes beating rate on iron uptake, however, requires further studies for its clarification. Thirdly, although HL-1 cells are the only cardiomyocyte cell line that retains contractile phenotype with differentiated cardiac characteristics [25], [26], they are established from AT-1 mouse atrial cardiomyocyte tumor lineage. The different electrical properties, including calcium kinetics, between atrial and ventricular myocytes [52] may potentially lead to differences in response to iron overload between HL-1 cells and ventricular cardiomyocytes merit further studies. With advances in the induced pluripotent stem cell technology, the use of human ventricular cardiomyocytes may be a better model to study the effects of iron cardiotoxicity. Finally, we have not assessed the detailed pro-apoptotic signaling pathways in the present study. In mesenchymal stem cells [59], [60], hepatocytes [61], neuroblastoma cells [62] and gerbil [63], p38 and JNK are activated under iron overload conditions. This would undoubtedly be important when designing future studies.

## Conclusions

In summary, our current study illustrated the patterns of iron entry in HL-1 atrial myocytes under ferric or ferrous iron overload condition. The blockade of LTCC but not TTCC was identified to prevent labile ferric iron entry. The uptake of ferrous iron probably involves other mechanism. As expected, iron overload was shown to induce cardiac apoptosis via mitochondria-mediated caspase-3 dependent pathways. However, LTCC blockers have very limited protective effect toward iron induced apoptosis. Our study provided a better understanding to the role of LTCC and TTCC on NTBI uptake into cardiomyocytes, contributing to the conceptual framework in the development of advanced therapeutic strategy for IOC in combination with the current chelation therapy.
